# Patient participation in medical student teaching: a survey of hospital patients

**DOI:** 10.1186/s12909-020-02052-1

**Published:** 2020-05-07

**Authors:** Nathan G Rockey, Guilherme Piovezani Ramos, Susan Romanski, Dennis Bierle, Matthew Bartlett, Magnus Halland

**Affiliations:** 1 Mayo Clinic Alix School of Medicine, 200 1st SW, Rochester, MN 55905 USA; 2 Division of Gastroenterology and Hepatology, Mayo Clinic, 200 1st SW, Rochester, MN 55905 USA; 3 Department of Internal Medicine, Mayo Clinic, 200 1st Street SW, Rochester, MN 55905 USA

**Keywords:** Undergraduate medical education, Inpatient teaching, Bedside teaching

## Abstract

**Background:**

Despite the common practice of involving in-patients in the teaching of medical students little is known about the experience for patients. This study investigated inpatients' willingness, motivations and experience with participation in medical student bedside teaching.

**Methods:**

In-patients at a tertiary hospital who participated in medical student teaching answered a 22 question survey. The survey examined the motivations, impact and overall experience for these patients.

**Results:**

During July and August of 2019, 111 patients aged 19–93 years completed the survey. Most patients who were approached by preceptors to participate in teaching agreed to participate (74%). Ninety-six percent of patients felt like they could have said no if they had not wanted to participate in medical student teaching. Ninety percent of patients valued the time they spent with students.

**Conclusions:**

Most hospital inpatients are willing to participate in medical student teaching in order to be helpful, and most have a positive experience. Preceptors in undergraduate medical education should prioritize a quality informed consent process and understand that the teaching experience can be mutually productive for patients and students.

## Background

Knowing how to obtain a medical history and perform a comprehensive physical exam are key skills that medical students need to learn in preparation for clinical practice. This learning occurs via a variety of modalities including didactic lectures, simulation center sessions, and patient encounters. Simulation sessions offer numerous benefits and are opportunities to practice and fail in a defined encounter that is outside of the real patient care setting. While this technology has continued to improve, real patient encounters are still considered an important component of the early stages of undergraduate medical education [1–3]. In fact, early clinical exposure has increasingly been studied as a way to help students transition from the classroom to the wards [4–6].

Patients in an outpatient setting generally have a positive outlook on interacting with students [7–16]. While prior work has shown positive patient experiences in the outpatient setting, little is known about patients’ experiences interacting with medical students in the hospital. Interactions between medical students and patients in the hospital bring unique considerations to the educational experience, including the severity of patients’ illnesses, the potential for interfering with the flow of the hospital stay including team rounds, and other needs of the patient such as sleep and family visitation. Students have described not wanting to burden the patient as a barrier to participating in bedside teaching [17]. However, it is not clear whether patients in the hospital view interactions with medical students as a burden. The data on patients’ perspectives on participating in medical student or junior medical staff teaching in the in-patient setting is limited [18–21]. For example, Nair et al. [18] found that patients generally had a positive outlook on participating in bed-side teaching with junior medical trainees including interns, residents and medical students, but only 37.5% of the learners in the study were medical students. Only two prior studies have focused specifically on medical students in the hospital and combined included only 150 patients who had interacted with a student, of which 73 were from a geriatric population. The 73 geriatric patients generally had a positive outlook on in-hospital participation in medical student teaching, although 9 and 23% of patients noted the process was tiresome or time-consuming respectively [19]. Furthermore, in a survey of 77 patients who either independently or with assistance of the medical student filled a 4 page survey, only 50% patients felt that previous interactions with medical students positively influenced their time in the hospital [20]. None of these studies focused specifically on medical students outside of the patient care team. This is important because it is possible that there is a difference in willingness to participate in teaching when the medical student is on your care team, as patients might perceive their participation is indirectly implicated in the quality of the care they receive.

Taken together, the data from the outpatient studies found that patients have a positive experience with participating; however given the increased acuity of illness it is unclear whether inpatients will have the same experience. The motivation for inpatient participation in medical student teaching is hitherto largely unexplored, and it has not been established whether inpatients feel a sense of obligation to teach students. It is important to determine whether inpatients may feel coerced to participate while they are sick and vulnerable in the hospital. Thus, more data are needed to understand patients’ experience with participating in undergraduate medical education in the hospital. In this study we hypothesized that a majority of patients would be interested in participating in medical student teaching and that this experience would be overall positive. Therefore, the aims of this study were to understand the experience and motivations of hospital inpatients who participated in medical student teaching during their hospitalization.

## Methods

### Patients

Patients aged 18 and above who were admitted to medical or surgical wards in a large tertiary referral teaching hospital were eligible to participate in a survey about their experience. A physician preceptor in their role as medical school faculty member, who was not part of the care team, approached patients for medical student teaching as a standard part of the second year medical student curriculum. All patients gave verbal informed consent to participate in teaching in accordance with institutional guidelines and preceptors were asked to record the percentage of patients who agreed to participate in teaching. Patients were then asked to participate in the optional survey. Patients who agreed to study participation and completed the survey were compensated USD 25. Pediatric patients as well as patients with altered mental status and inability to provide informed consent were excluded from study participation. This study was approved by the Mayo Clinic Institutional Review Board.

### Medical student teaching session

Patients participated in either a small group preceptor-led bedside teaching session focusing on physical examination skills or an individual student session in which a student completed a history and physical exam. These teaching rounds are separate from in-patient care team teaching, and are led by a medical school preceptor who is typically a senior physician. In the bedside teaching sessions, three to four students and one physician preceptor spent 15–30 min with a patient who had one or more pertinent physical exam findings. In the individual student session, a second year medical student spent up to an hour with a patient gathering a history and completing a physical exam.

### Assessing willingness to participate

Preceptors were asked to record how many patients they approached in the hospital to participate in the teaching experience and how many of these patients agreed to participate after the informed consent process.

### Survey

Study participants were asked to fill out a two page survey immediately after taking part in the teaching experience. The survey was developed by M.H. and N.R. after reviewing the questions asked of patients in previous studies that assessed their experiences [8, 10, 13, 18–20, 22] and creating questions that answered specific components of our hypothesis: factors that may affect motivation for participation (adequacy of informed consent, desire to teach students, sense of obligation, boredom), and examination of the quality of the experience (effect on happiness, pain, perception of hospital stay). The survey included 19 statements that were rated by patients on a Likert scale from 1 to 5, and three short answer questions which allowed for free-text answers and comments. The three short answer questions were (1) “Why did you participate in the teaching experience?”, (2) “What was the best part of participating in teaching?” and (3) “What was the worst part of participating in teaching?” The survey was filled independently by the patient and not in the presence of either the preceptor or medical student and placed in sealed envelope without any identifying information. The sealed envelope was collected within 24 h of the teaching encounter and the preceptors and medical students who interacted with a specific patient did not have access to any patient’s individual responses.

### Data analysis

Responses to the short answer questions were first coded with descriptive terms using an open coding method and reviewed by the investigators. This content analysis [23] was felt to be sufficient given the brevity of short answer responses and no further steps of coding were conducted. The following general demographic data were also collected: age, gender, indication for being in hospital and length of hospital stay. The results of the survey were compiled using SAS software (JMP Pro 14.1.0) to analyze the data. Answers to specific questions 8, 9, 12, 13, 14, and 15 were converted to a binary variable (agree or not, for those above or equal to 4). Then, the effect of the desirable factors/variables (Age, gender, length of hospital stay and teaching type) were assessed in a 2 by 2 table with *p* value obtained utilizing Fisher Exact Test. All hypothesis tests were two-sided and *p*-values of less than 0.05 were considered statistically significant.

## Results

### Demographic results and survey completion

A total of five preceptors collected 111 surveys spread across 7 days of teaching rounds between July and August 2019. Likert scale responses are presented in Table 1. Demographic data are presented in Table 2, and 56 (51%) of patients were female. The mean age was 63 (range 19–93 years), and 55 patients (50%) were aged 65 or above. Fifty-four patients participated in the preceptor-led bedside teaching sessions and 54 in the history and physical examination session with a single medical student. The median length of stay at the time of the medical student interaction was 4 days.

**Table 1:** Survey with Likert Scale Responses

Questions*	**Strongly Disagree % (n)**	**Disagree % (n)**	**Neutral % (n)**	**Agree % (n)**	**Strongly Agree % (n)**
**Motivation to participate**					
1. I felt comfortable with being asked to participate in medical student physical exam/history taking teaching.	1 (1)	1 (1)	1 (1)	16 (18)	81 (89)
2. I felt like I could have said no if I had not wanted to participate in medical student teaching.	(2)	0 (0)	2 (2)	15 (16)	82 (90)
3. I understood what the experience would be like before saying yes	1 (1)	2 (2)	9 (10)	26 (29)	62 (68)
4. Participation in this teaching has no impact on the care I receive while in the hospital.	4 (4)	2 (2)	5 (5)	18 (20)	72 (80)
5. It was clear that the medical students were not part of my care team.	0 (0)	1 (1)	4 (4)	13 (14)	83 (91)
6. I have a good understanding of why I am in the hospital, and what my diagnosis is.	0 (0)	1 (1)	4 (4)	26 (29)	69 (76)
7. If patients don’t teach doctors in-training, they won’t be able to learn what they need to learn.	1 (1)	5 (5)	6 (7)	23 (25)	65 (72)
8. Patients have a duty to teach when they are in the hospital.	6 (6)	18 (20)	24 (26)	23 (25)	29 (32)
9. Patients should expect to participate in teaching when they enter the hospital.	5 (6)	21 (23)	26 (29)	22 (24)	25 (28)
**Quality of Experience**					
10. The medical students treated me with respect.	0 (0)	1 (1)	0 (0)	7 (8)	92 (101)
11. I value the time spent with medical students.	0 (0)	0 (0)	10 (11)	21 (23)	69 (77)
12. I learned something about my health/disease by participating in this teaching.	2 (2)	8 (9)	35 (39)	20 (22)	35 (39)
13. Participation in this teaching made me happier	0 (0)	2 (2)	20 (22)	34 (38)	44 (49)
14. Participation in this teaching made me feel better about my hospital stay.	1 (1)	1 (1)	23 (25)	41 (45)	35 (39)
15. I would participate in this teaching again.	1 (1)	0 (0)	2 (2)	25 (28)	72 (79)
**Question**	**None % (n)**	**A slight amount % (n)**	**A moderate amount % (n)**	**A lot % (n)**	**A huge amount % (n)**
**Quality of Experience**					
16. How much pain were you in while the medical students were with you?	52 (57)	28 (31)	13 (14)	6 (6)	1 (1)
17. Did taking part in the teaching worsen your pain?	97 (105)	2 (2)	1 (1)	0 (0)	0 (0)
**Motivation to participate**					
18. How much do you feel like patients have a duty to teach?	17 (18)	18 (20)	25 (27)	18 (20)	22 (24)
19. How bored were you when the doctor came in to ask you to participate in teaching?	75 (82)	9 (10)	13 (14)	2 (2)	1 (1)

*Complete survey divided between questions that assessed motivation to participate and quality of experience. Percentages of responses to each question are reported with the absolute number of responses in parentheses. There were 111 total surveys filled out

**Table 2:** Demographic Data

Gender	Number of patients % (n)
Female	50 (56)
Male	48 (53)
Unspecified	2 (2)
**Years**	
19–39	6 (7)
40–49	12 (13)
50–59	19 (21)
60–69	26 (29)
70–79	22 (24)
80–89	14 (16)
90–99	1 (1)
**Diagnosis by System**	
Cardiac	39 (43)
GI	28 (31)
Pulmonary	12 (13)
Nephrology	5 (6)
Neurology	5 (6)
Other/unspecified	11 (12)
**Days in Hospital**	
1–4	64 (68)
5–9	22 (23)
10 or more	14 (15)
**Teaching Type**^**a**^	
Bedside teaching group	49 (54)
Individual student	49 (54)
Both	1 (1)
Unspecified	2 (2)

^a^percentages round up to 101 due to rounding

### Willingness to be involved in teaching

Four out of five preceptors recorded data on willingness to participate in teaching. This subset of preceptors approached 145 patients, of whom 107 (74%) agreed to be a part of the second year medical student teaching.

### Survey results

Overall, 99% of the Likert scale questions were completed, and 94% of the short answer questions were filled (Table 1). One patient participated in both sessions on separate days and data for type of teaching were missing for two patients.

### Patients motivations for participating in teaching

Ninety-seven percent of patients (107/110) felt comfortable with being asked to participate in the teaching (Table 1). 96% Ninety-six percent of patients (106/110) felt like they could have said no if they had not wanted to participate in medical student teaching. Three percent of patients (3/110) reported not understanding what the experience would be like before saying yes. Fifty-two percent of patients (57/109) agreed or strongly agreed to the statement, “Patients have a duty to teach when they are in the hospital”, while 24% (26) disagreed or strongly disagreed. Forty-seven percent of patients (52/110) agreed or strongly agreed to the statement, “Patients should expect to participate in teaching when they enter the hospital”.

Eighty-eight percent of patients (97/110) agreed or strongly agreed to the statement, “If patients don’t teach doctors in-training, they won’t be able to learn what they need to learn”, while (5%) (6/110) disagreed or strongly disagreed. Seventy-five percent of patients (82/109) reported that they were not bored when they were asked to participate. In response to the short answer question “Why did you participate in the teaching experience?”, 71% of patients (76/107) referenced helping students, future patients or both. These include, “*How else can they learn? Seeing things sticks with you easier”* and *“I believe in helping when I’m needed. It’s for the good of all patients.”* (Table 3).

**Table 3:** Coding of short answer responses with corresponding examples and number of responses

Code*	Example	Number responses % (n)
*A. Why did you participate in the teaching experience?*		
**To help student learn**	“to help those learning to be doctors understand human connection”	52 (56)
**To help future patients**	“hopefully what the student learns will help someone else”	7 (7)
**Combination of 1 and 2, being helpful in general**	“help future drs and patients”	12 (13)
**To learn from student**	“to try and learn about my condition. It was explained to me very well. All questions were answered very well”	7 (7)
**Fun, bored, passing time**	“it looked interesting”	7 (8)
**Giving back to institution**	“this has been a large part of mayo and its history - it is a learning institution”	3 (3)
**Opportunity to talk**	“I had a good story to share”	4 (4)
“**Because they asked”**	“I was politely asked”	7 (8)
“**Right thing to do”**	“it was the right thing to do”	1 (1)
*B: What was the best part of participating in teaching?*		
**Helping student learn**	“helping the students learn about different medical issues”	38 (39)
**Helping future patients**	“knowing they can help others and feel comfortable with patients	1 (1)
**Combination of 1 and 2, being helpful in general**	“Knowing that I am helping someone learn their passion of becoming a doctor someday and to know that they will help another patient in their care and saving someone’s life someday”	12 (12)
**Being part of process, seeing students grow**	“seeing how interested the student was in my medical problems”	15 (16)
**Opportunity to talk and be vulnerable**	“feeling like I could be honest”	3 (3)
**Learning from student**	“I learned more about the symptoms of my disease”	14 (15)
**Giving back to institution**	“Knowing they were being trained at an excellent hospital”	2 (2)
**Vague/not specifically coded**	“all of it”	14 (15)
**Break from boredom**	“the conversation broke up the monotony”	1 (1)
*C: What was the worst part of participating in teaching?*		
**None**	“nothing! It was great and I’m happy to help”	88 (97)
**Difficult to share/be the subject**	“having to talk about/go over what happened again” “Feeling like in a zoo (observation)” “Discussing my issues with alcoholism and bulimia”	5 (6)
**Difficult environment to teach in**	“that it was interrupted due to procedures”	2 (2)
**Nervous**	“I was nervous”	1 (1)
**Negative experience with a student**	“one student was very assertive in his touch, almost causing some pain as he poked/prodded”	1 (1)
**Too long**	“a little long”	1 (1)
**Tired**	“I was tired, it was late”	1 (1)
**Not long enough**	“the student only asked 1 question”	1 (1)

*Codes for each short answer questions are shown with one specific example. In the cases where there was only one response for the code, the example given is that response. Otherwise, a response that exemplifies the code was chosen

### Quality of experience

**Fig. 1:**
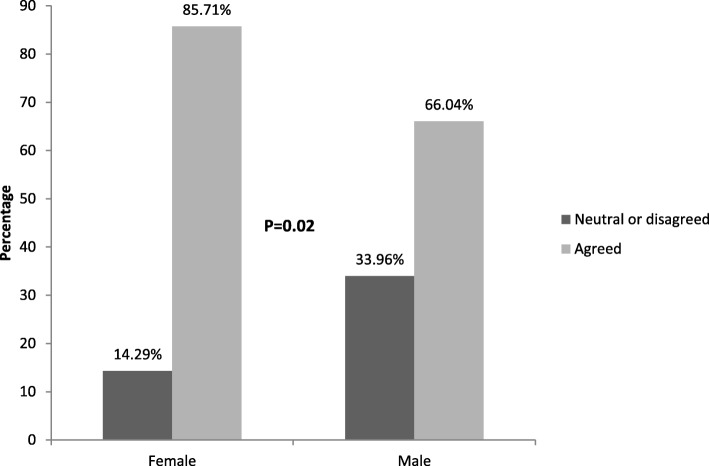
Responses to the statement, “Participation in this teaching made me feel better about my hospital stay” based on gender. *Responses of “Strongly agree” and “agree” were grouped together. Responses of “Strongly disagree”, “disagree”, and “neutral” were grouped together. Female patients were more likely to agree with the statement than male patients

Forty-eight percent of patients (52/109) reported they were in pain at the time of the teaching experience. Of these patients who reported pain, 60% (31) reported slight pain and 40% (21) reported moderate to severe pain. Three percent of patients (3/108) said that taking part in the teaching worsened their pain to some extent. Ninety percent of patients (100/111) valued the time they spent with medical students. One patient would not participate in the teaching again and two were neutral. Seventy-eight percent of patients (87/111) said that participating in teaching made them happier, and there was no difference when analyzing age, gender, length of hospital stay and teaching type. Females more commonly reported agreeing that participating made in the session made their hospital stay better (86% vs 66%) *P* = 0.02. (Fig. 1).

Fifty percent of patients (52/104) said that the best part of teaching was helping students, helping future patients, or being helpful in general (Table 3). Fifteen percent of patients (16/104) said the best part of the experience was related to the process and seeing students grow (Table 3). For example, one patient said, “seeing how interested the student was in my medical problems”. Fifteen percent of patients (15/104) said the best part of the experience was learning from the student (Table 3).

**Fig. 2:**
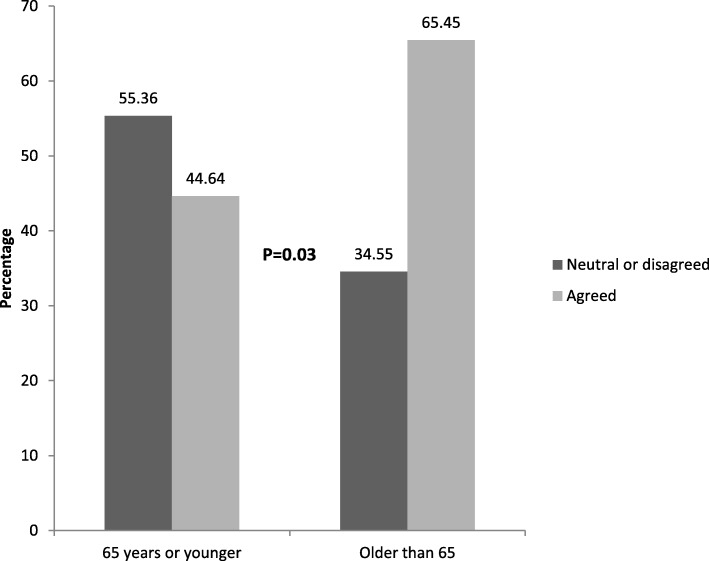
Responses to the statement, “I learned something about my health/disease by participating in teaching” based on age. *Responses of “Strongly agree” and “agree” were grouped together. Responses of “Strongly disagree”, “disagree”, and “neutral” were grouped together. Patients older than 65 were more likely to agree with the statement than patients 65 years or younger

Eighty-eight percent of responses (97) to “What was the worst part of teaching” indicated that there was no worst part. Five percent of responses (6) to this question were coded as “difficult to share/be the teaching subject”. These included specific patient comments such as: *“Having to talk about/go over what happened again”, “Feeling like in a zoo (observation)” “Discussing my issues with alcoholism and bulimia”.* Four percent of responses (4) included information about a negative experience: “the *process took too long”, “I felt nervous”, “I was too tired”* and *“being poked and prodded with the student causing pain”.* Fifty-five percent of patients (61/111) felt that they learned something about their health/disease by participating in this teaching, 35% (39/111) were neutral, and 10% (11/111) disagreed or strongly disagreed. Patients 65 years and older agreed with having learned something from the experience more frequently than when compared to younger patients less than 65 years old (65% compared to 44% *P* = 0.03) (Fig. 2).

## Discussion

This is the first study to our knowledge that specifically examined in-patients experience with participating in preceptor led teaching sessions with medical students who were not part of the patient care team. There are three principal findings of our study; (1) nearly three quarters of hospital inpatients who are asked to participate in medical student teaching are willing to do so, (2) the majority of patients who agree to participate value the time spent with students and would participate again, and (3) most patients who agree to participate in teaching agree to do so because they want to be helpful.

It was remarkable to note that 108 of 110 patients did not feel pressure to participate in the teaching, reflecting that the autonomy of patients is being respected in the consent process for teaching at our program. However, 3% of patients reported that they did not understand what the teaching session would involve, and this highlights an opportunity for improvement with the aim that all patients who volunteer to take part in medical student teaching do so with a full understanding of what the teaching session entails.

This study helps fill a gap in the literature by asking patients in the hospital why they participate in medical student teaching. Being helpful to students and future patients was the most common motivation for agreeing to participate. Many patients who agree to participate are aware of their contribution to student teaching, and highlights the role they see for themselves in the teaching process – being a real-life example of what students are learning about in the classroom. Patients also referenced helping future patients. In this conceptualization, medical students are a part of a broader goal, in which patients hope to improve the lives of other patients in the future. The language of expectation or sense of duty did not broadly resonate with patients in the way that other statements on the survey resonated. However, only three patients did not express a willingness to participate in the teaching again. It is likely that patients agree to participate out of a sense of generosity and altruism, rather than a sense of obligation [24]. We argue that the profession must appreciate and acknowledge the altruism that is involved when patients, in times of suffering, teach medical students.^1^

In a prior study of outpatients and inpatients, female patients were more likely to have a negative attitude towards medical students involved in their care than male patients [22] While our study is about incorporating teaching into the hospital experience and specifically not about care – it is important to note that the only gender difference our study elucidated was that females were more likely to agree that participating made their hospital stay better.

While the majority of responses indicated that patients viewed the interaction with the medical student as a positive experience, there were exceptions. While rare in our cohort, these responses raise important questions. Is it ethical to make patients describe their often difficult health journey for students when that process of sharing is not directly related to their care? It might be argued that this risk must be balanced with the potential beneficial effect of having the opportunity to talk with students. For example, more than half of our patients said that participation made them happier. Regardless, students and preceptors need to be aware of the possibility that bedside teaching can be challenging for patients and be mindful of these issues during teaching sessions to maintain patient autonomy and dignity. More research is needed to delineate this balance. Only one of 110 patients said the worst part of the teaching was that it was too long, while Aquilina et al. found that 23% of patients said their experience was time consuming [19].

Ensuring that medical education does not interfere with patient care is an important concern, and there are data that suggest that patients want students roles on medical care teams to be made more clear [25]. While our study was strictly about education and not about medical students as observers or participants in medical care, most patients in our study said they had an understanding of what the process was going to be like before it started, and the vast majority indicated they were aware the medical student was not part of the care team. This finding is in stark contrast to a survey of 100 patients who participated in bedside teaching where 63% said they were not properly forewarned [18]. This highlights the importance of a thorough informed consent process, which should always be incorporated into medical student teaching in the hospital regardless of whether the medical student is part of the care team.

Whilst moderate or severe pain was uncommon in our sample overall, 41% of patients did report some level of pain during the teaching session. Preceptors hope to select patients with physical exam findings for bedside teaching, but it is also important to not overly burden an acutely sick patient [26]. Importantly, most patients in this study reported that participating did not worsen their pain. The principle of “first, do no harm” must also apply to medical education in the hospital.

Strengths of this study include that it was focused solely on the patients experience with a defined medical student encounter as opposed to medical student participation and teaching as part of a clinical care team and included more than 100 patients. In addition, the preceptors were also not involved in the clinical care of the patient, which serves to prevent any conflict of interest and undue pressure to participate. Furthermore, the patients all filled in their own survey without assistance or presence of preceptors, study staff or medical students and placed anonymous responses in a sealed envelope thus lessening the chance of bias. Additionally, unlike other surveys in the literature [19, 20, 22], this study asked patients about a specific teaching experience directly after it happened, rather than surveying patients in general about their attitudes towards medical student’s involvement in patient care or collecting information about the teaching experience as part of a discharge questionnaire. Our survey was brief and patients overwhelmingly were able to complete the entire survey leading to little missing data.

The study also has several limitations. Because our study was specific to medical education, our results might not apply to medical student involved in the care team. While preceptors who recruited patients were not part of the care team and we made an effort to make this clear, there still exists a power differential between physicians and patients which might affect the percentage of patients who are willing to participate. Thus, patients’ perception of the survey may be impacted if they do not distinguish between clinicians doing research and clinicians who are on their care team.

Four out of five preceptors recorded the number of patients who declined participation in medical student teaching, and hence data was missing from one preceptor which is a limitation regarding the data on willingness to participate. Also, the true survey response rate cannot be calculated as we did not record how many patients agreed to participate in teaching, but declined study participation, although it was universally felt among the preceptors that the vast majority of patients who consented to teaching also consented to study participation.

Similar to other survey based studies of the patient experience [19, 20, 22] we did not use a validated tool as none is currently available. N.R. and M. H designed the survey and reviewed the questions for clarity and relatedness to the hypothesis, however, formal pilot testing did not occur. Our study was primarily descriptive and given the small sample size and nature of our survey, a robust statistical analysis was not possible. This study only includes patients who agreed to teaching, thus factors which contribute to non-participation in teaching in the hospital setting remain unexplored. Younger patients were underrepresented in our study – while the median age was 64, there were only 7 patients less than 40 years old. Finally, this study was conducted at a large academic hospital and may not be applicable to other settings.

Future research should seek to understand reasons for not participating in teaching. An analysis of factors that contribute to patients not wanting to be involved in teaching would be helpful for preceptors to know which patients to ask about teaching participation. Based on our findings we recommend the following guiding principles for in-patient bedside teaching; (1) the teaching process including the expected time-commitment needs to be clearly outlined, (2) the role of the student needs to be unambiguously presented and (3) careful consideration of the impact of pain, particularly during physical examination teaching is required. When these criteria are met, true informed consent can be obtained and the patient-student interaction is likely to be a bilaterally positive experience. Preceptors should prioritize creating a setting where these factors are present and always respect the autonomy of patients, and that while they may be in a teaching hospital, it is not their obligation to be involved in medical student education. Ideally, when the above conditions are met, inpatient participation in medical student teaching can be a positive and uplifting component of a patient’s hospital stay, rather than an unwelcome intrusion. Our data suggest that this is a reasonable goal.

## Conclusions

Bedside teaching and interactions with patients in the hospital are essential components of undergraduate medical education. This study suggests that most patients who agree to participate in medical student education have a positive experience and that patients agree to be teaching subjects because they want to help. Indeed, patient participation in medical education is not an obligation or something to expect, but rather an intentional expression of altruism.

## Data Availability

All data generated or analyzed during this study are included in this published article.

## Footnotes

1 Rockey N. “Recognising the Generosity of Patients” The BMJ Opinion 2019. Available from: https://blogs.bmj.com/bmj/2019/05/07/nathan-rockey-recognising-the-generosity-of-patients/.
